# Measurement of brain lactate during visual stimulation using a long TE semi‐LASER sequence at 7 T

**DOI:** 10.1002/nbm.4223

**Published:** 2020-01-29

**Authors:** Carolina C. Fernandes, Bernard Lanz, Chen Chen, Peter G. Morris

**Affiliations:** ^1^ Sir Peter Mansfield Imaging Centre University of Nottingham Nottingham, NG7 2RD Nottinghamshire United Kingdom

**Keywords:** functional MRS, lactate, long TE, macromolecules, semi‐LASER

## Abstract

Estimation of metabolic changes during neuronal activation represents a challenge for in vivo MRS, especially for metabolites with low concentration and signal overlap, such as lactate. In this work, we aimed to evaluate the feasibility of detecting lactate during brain activation using a long TE (144 ms) semi‐LASER sequence at 7 T. ${}^{1}H$ spectra were acquired on healthy volunteers ( N=6) during a paradigm with 15 min of visual stimulation. Outer‐volume signals were further attenuated by the use of saturation slabs, and macromolecular signals in the vicinity of the inverted lactate peak were individually fitted with simulated Lorentzian peaks. All spectra were free of artefacts and highly reproducible across subjects. Lactate was accurately quantified with an average Cramér‐Rao lower bound of 8%. Statistically significant ( P<0.05, one‐tailed t‐test) increases in lactate ( ∼10%) and glutamate ( ∼3%) levels during stimulation were detected in the visual cortex. Lactate and glutamate changes were consistent with previous measurements. We demonstrated that quantification of a clear and non‐contaminated lactate peak obtained with a long TE sequence has the potential of improving the accuracy of functional MRS studies targeting non‐oxidative reaction pathways.

## Graphical abstract

Lactate and glutamate changes during a paradigm with 15 min of visual stimulation were measured with an optimized J‐modulation selection protocol ( TE=144ms) to increase the sensitivity of lactate detection. This novel functional MRS approach resulted in a reliable lactate peak fit arising from the clean baseline and the inclusion of neighbouring macromolecule components in the basis set. We showed that a long TE sequence has the potential of improving the accuracy of functional MRS studies targeting non‐oxidative reaction pathways.

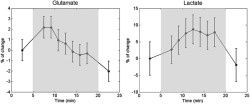

**Abbreviations**
CRLB: Cramér‐Rao lower bound
fMRS: functional MRS
Glu: glutamate
Gln: glutamine
IR: inversion recovery
Lac: lactate
MM: macromolecules
VOI: volume‐of‐interest.

## 1 INTRODUCTION

The study of metabolic fluctuations during neuronal stimulation has been a topic of interest since Fox and colleagues^1^ used PET to demonstrate the uncoupling between glucose consumption and oxidative metabolism. This mismatch is attributed to a rise in non‐oxidative metabolism during brain activation, manifest as an increase in the concentration of lactate (Lac),^2^ as a means to respond to the increased energy demand. Despite being a less energetically efficient process, the temporarily increased non‐oxidative reaction pathway can potentially generate ATP, the energy substrate for all cellular processes, through glycolysis faster than through full glucose oxidation in the tricarboxylic cycle. Lac is therefore a particularly interesting molecule in brain energy metabolism. The first measurements performed with in vivo MRS to determine Lac concentration changes in the primary visual cortex during visual stimulation detected rises in Lac levels of 57%,^2^ 170%^3^ and 250%,^4^ while a study by Merboldt et al^5^ reported an absence of Lac accumulation. Although the results of the initial studies were contradictory, consistent changes in Lac concentration upon visual stimulation have been reported at 7 T, benefitting from the increase in SNR and spectral resolution at ultra‐high field. The average rise in Lac level during visual stimulation was found to be in the range of 7% to 37%,^6^, ^7^, ^8^, ^9^, ^10^, ^11^, ^12^, ^13^ maintained until the end of the stimulus in most studies. However, these 7 T studies were generally designed to follow the time courses of all neurometabolites detectable by ^1^H MRS and were not specifically optimized for measurement of Lac. Due to the low concentrations of metabolites in normal brain tissue, the majority of functional MRS (fMRS) studies thus used short TE sequences, reducing both signal loss by transverse relaxation mechanisms and J‐modulation effects. With this method, the accurate determination of Lac, whose resonances overlap with higher amplitude signals, in particular those from macromolecules (MM) and lipids, can be challenging. Usually, the dissociation of the MM/lipid and Lac contributions is accomplished by linear fitting of model spectra. Even in brain tissue, where lipid contamination is less intense than in many other tissues, muscle for example, the robustness of this fitting depends on a priori knowledge of the MM profile and the spectral baseline. Spectral quantification with a general measured MM profile may not be sufficiently robust to account for inter‐subject variability in the different MM components. A previous study investigating whether tissue‐specific MM signal affects metabolite quantification showed that a general MM profile provides an accurate determination of most metabolites.^14^ However, significant differences in Lac levels were found between spectral quantification with a MM profile acquired from a predominantly white or grey matter rich tissue. Given that the metabolic changes during neuronal stimulation are relatively small, accurate quantification of the resonances of interest is imperative. The aim of this work was to develop a novel fMRS approach with an optimized J‐modulation selection (*TE* = 144 ms), thereby minimizing the contribution of overlapping signals to the Lac peak. We have used it to reliably investigate the metabolic changes during a prolonged visual stimulus of 15 min in Lac and other key metabolites, such as glutamate (Glu).

## 2 EXPERIMENT

### 2.1 MR measurements

**Figure 1:**
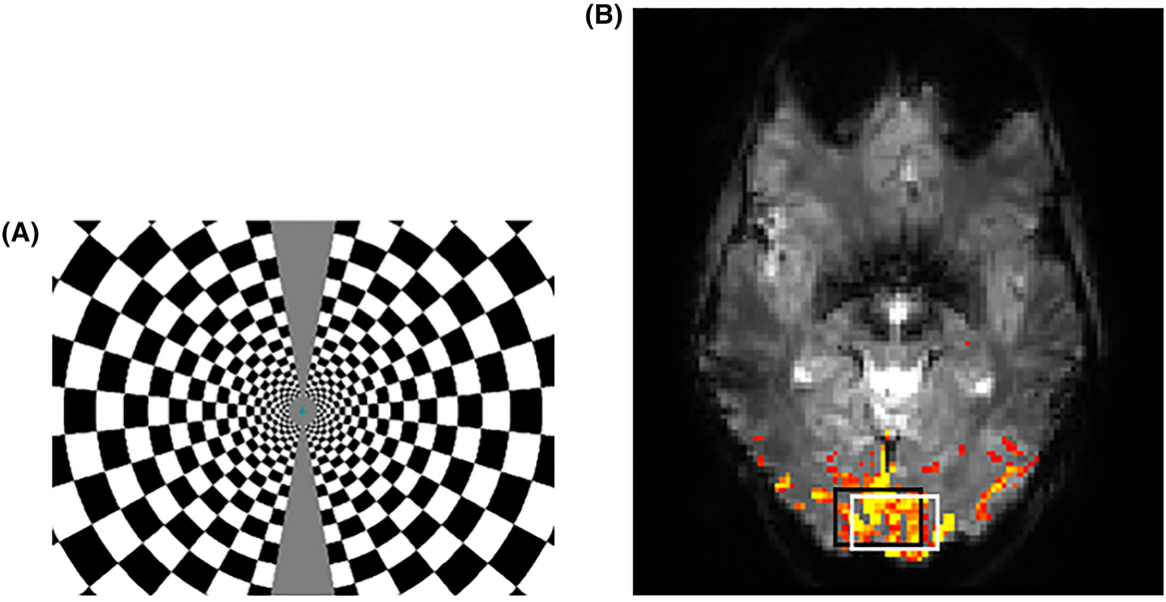
Positioning of the VOI. Assessment of neuronal activity following the application of the activation paradigm with a radial checkerboard pattern A, aided the spectroscopic voxel placement. The representative BOLD response B, shows the voxel position ( $20\times 30\times 20{\mathrm{mm}}^{3}$) in the activated visual cortex ( P=0.05). The Lac VOI (black box) is chemically shifted from the water VOI (white box)

Six healthy volunteers (three males; age range 25‐30 years) participated in this study and gave informed consent to a protocol approved by the local ethics committee. MR measurements were made on a 7 T Philips Achieva MR system (Philips Healthcare, Best, The Netherlands) with a 32‐channel receive head coil (Nova Medical, Wilmington, Massachusetts, USA) and a volume transmit coil (Nova Medical) with a maximum transmit $B_{1}$ field of 20 μT. Prior to the fMRS measurements, an fMRI localizer (multi‐slice EPI, TR/*TE* = 2000/25 ms, spatial resolution = $2\times 2\times 3{\mathrm{mm}}^{3}$, 30 slices) was performed to assist the positioning of the volume‐of‐interest (VOI) within the activated visual cortex. The activation paradigm consisted of 8 s of a radial checkerboard that reversed its contrast at 8 Hz, followed by 24 s of rest with a total of six repeats (Figure 1). High resolution anatomical images (MPRAGE, TR/*TE* = 7/3 ms, 1 ${\mathrm{mm}}^{3}$ isotropic resolution) were acquired to confirm the spectroscopic voxel position and to ensure that the VOIs ( $20\times 30\times 20{\mathrm{mm}}^{3}$) of water (4.7 ppm) and Lac (1.31 ppm) resonances were located entirely within the visual cortex. Water and Lac VOIs represent the limits of the chemical shift range including all resonances of the metabolites of interest. The fMRS measurements were performed using the same visual stimulus, with a paradigm that involved 15 min of stimulation (ON period), preceded and followed by 5 min of rest during which a uniform grey background was displayed (OFF periods). The ON period consisted of 50 s blocks of visual stimulation interleaved with short resting blocks of 10 s duration, in order to minimize adaptation and improve attention. To help maintain engagement, subjects were also asked to evaluate the colour of the fixation cross in the centre of the field‐of‐view and to respond via button press according to previous instructions.

${}^{1}H$ spectra were acquired during the stimulation paradigm for a total of 25 min using a semi‐LASER^15^ sequence ( TR/*TE* = 5000/144 ms). A broadband frequency‐modulated pulse with a bandwidth of 5372 Hz was used for excitation and two pairs of adiabatic full passage pulses with a bandwidth of 5424 Hz, centred on the water resonance, for refocusing. Water resonance suppression was performed with the VAPOR^16^ scheme and unsuppressed water spectra were also acquired before the acquisition of the dynamic spectra. A second‐order pencil‐beam volume shimming, a Philips routine based on FASTMAP,^17^ was applied to cover both water and Lac VOIs. To minimize contamination from extra‐cerebral lipid signals, a regional suppression technique^18^ was applied, by placing two saturation slabs onto the subject's scalp. To evaluate the contribution of the MM to the spectra at *TE* = 144 ms, metabolite‐nulled spectra were acquired using an inversion recovery (IR) semi‐LASER sequence ( TR/*TE* = 5000/144 ms, 64 scans) from a voxel with the same dimensions as previously described placed in the visual cortex of one subject. Four IR spectra were acquired with inversion times of 800, 900, 1000 and 1100 ms and one additional scan was performed without IR. Water suppression was achieved using the MOIST^19^, ^20^ technique, as the VAPOR option was not compatible with the pre‐existing IR module in the Philips pulse programming environment.

### 2.2 Data processing

The fMRS data acquired independently from the 32 channels were reconstructed based on an optimized coil combination method^21^ and frequency aligned using the Cr peak at 3.02 ppm in MATLAB (MathWorks, Natick, Massachusetts, USA). For each subject, spectra were averaged into blocks of 20 scans, followed by a moving average with a kernel of three blocks, resulting in nine spectra for further quantification (60 averages each). Only averages fully composed of blocks from either the ON or OFF period were kept, to avoid a mixture of signals acquired under different conditions.

**Figure 2:**
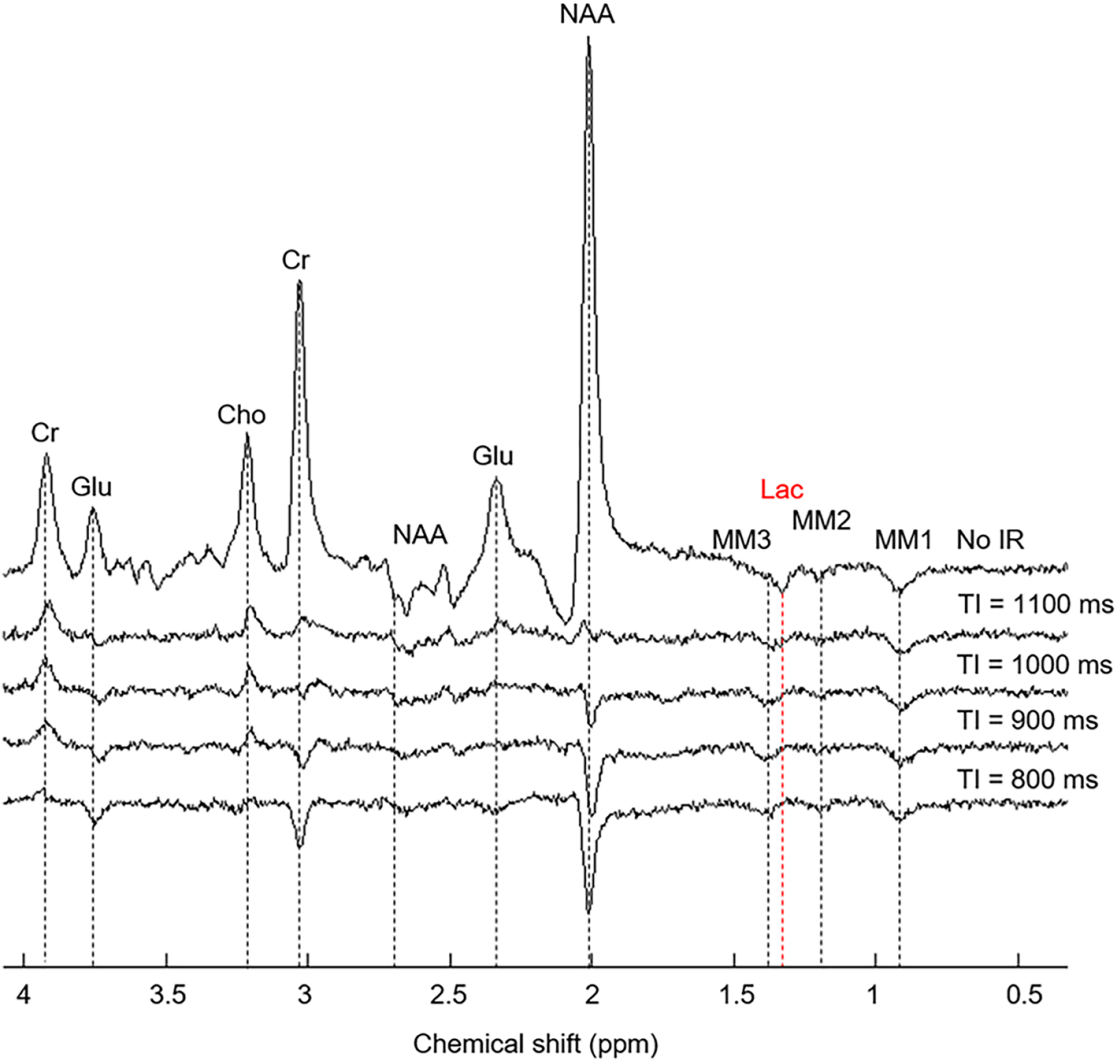
Identification of the main MM resonances. Spectra were measured with the IR technique, with inversion times of 800, 900, 1000 and 1100 ms. An additional spectrum without IR was also acquired (top). Spectra were obtained with a semi‐LASER sequence ( TR/*TE* = 5000/144 ms, 64 scans) from a $20\times 30\times 20{\mathrm{mm}}^{3}$ voxel placed in the visual cortex. Residuals from the metabolite‐nulling technique are visible in the IR spectra, as well as non‐nulled MM resonances MM1, MM2 and MM3

In addition, spectra were averaged across subjects after frequency alignment using the Cr peak, resulting in a time series with the same temporal resolution as previously, with each time point corresponding to a total of 360 averages (6 subjects × 60 averages ‐ group spectra). Spectra were quantified using LCModel,^22^ with a basis set composed of 19 metabolites, simulated using VESPA^23^ software: aspartate, Cho, Cr, GABA, glucose, Glu, glutamine (Gln), glutathione, glycerophosphocholine, glycine, Lac, myo‐inositol, NAA, N‐acetylaspartylglutamate, phosphocholine, PCr, phosphorylethanolamine, scyllo‐inositol and taurine. The methyl groups of NAA, Cr and PCr were added to the basis set separately to take into account their different transverse relaxation times.^24^ By visual inspection of the measured IR spectra (Figure 2), the residual MM resonances were identified, and consist of MM1 (0.89 ppm), MM2 (1.20 ppm) and MM3 (1.37 ppm). Note that MM1‐3 appear as inverted peaks at *TE* = 144 ms due to a homonuclear J‐evolution effect, as their J‐coupling constants (6.9‐7.3 Hz)^25^ are almost identical to that of Lac (6.9 Hz). These MM resonances were simulated in MATLAB as Lorentzian peaks of 3 Hz linewidth, to match the linewidth and shape of the simulated metabolite peaks, and then included in the basis set. LCModel analysis was performed over the spectral range from 0.2 to 4.2 ppm, with the node spacing of the spline function for baseline fitting (parameter dkntmn) set to 4 ppm, to ensure a flat baseline. The MM and lipid signals are strongly attenuated at a *TE* of 144 ms, as shown in Figure 2, leaving just the residuals MM1‐3 identified above. No additional MM or lipid resonances simulated by LCModel were added to the analysis. Metabolite levels were normalized to the unsuppressed water spectra. Statistical analysis was performed using a one sample t‐test on the percentage change of each time point of the individual subject metabolite time courses relative to the first OFF period (baseline). Glu and Lac time courses were analysed with a one‐tailed test, as a concentration increase is expected with stimulation. Additionally, NAA and total creatine (Cr and PCr) time courses were tested for significance with a two‐tailed test to assess the effect of the stimulus on metabolites whose levels are not expected to change.

## 3 RESULTS

**Figure 3:**
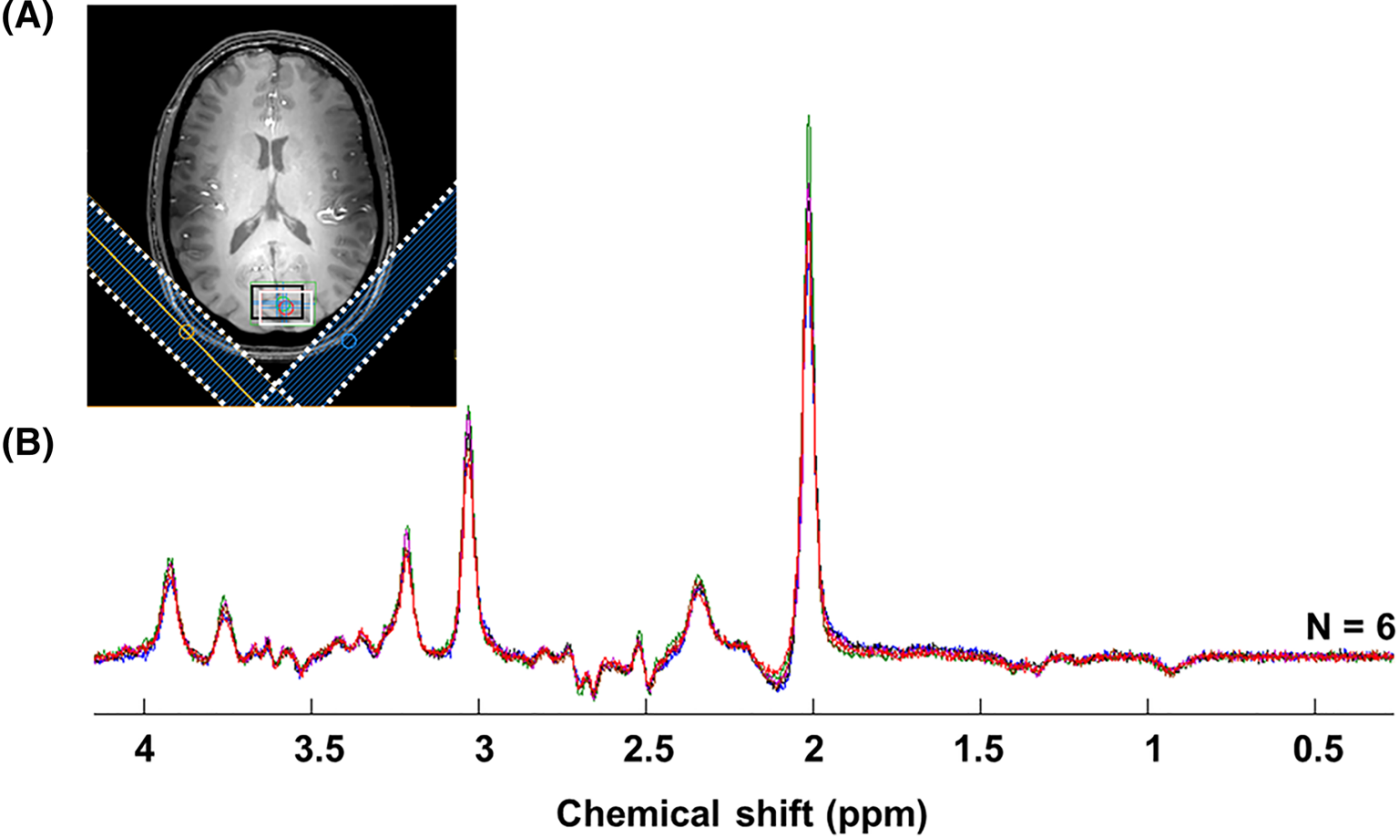
Robustness and reproducibility across subjects. Individual subject spectra ( N=6) were acquired from a voxel positioned in the visual cortex A, during the applied paradigm and summed over the entirety of the time course (540 averages for each spectrum). Spectra B, were measured with a semi‐LASER sequence ( TR/*TE* = 5000/144 ms) with lipid suppression applied by two saturation slabs (shown as dotted lines in A). The Lac VOI (black box) is chemically shifted from the water VOI (white box)

**Figure 4:**
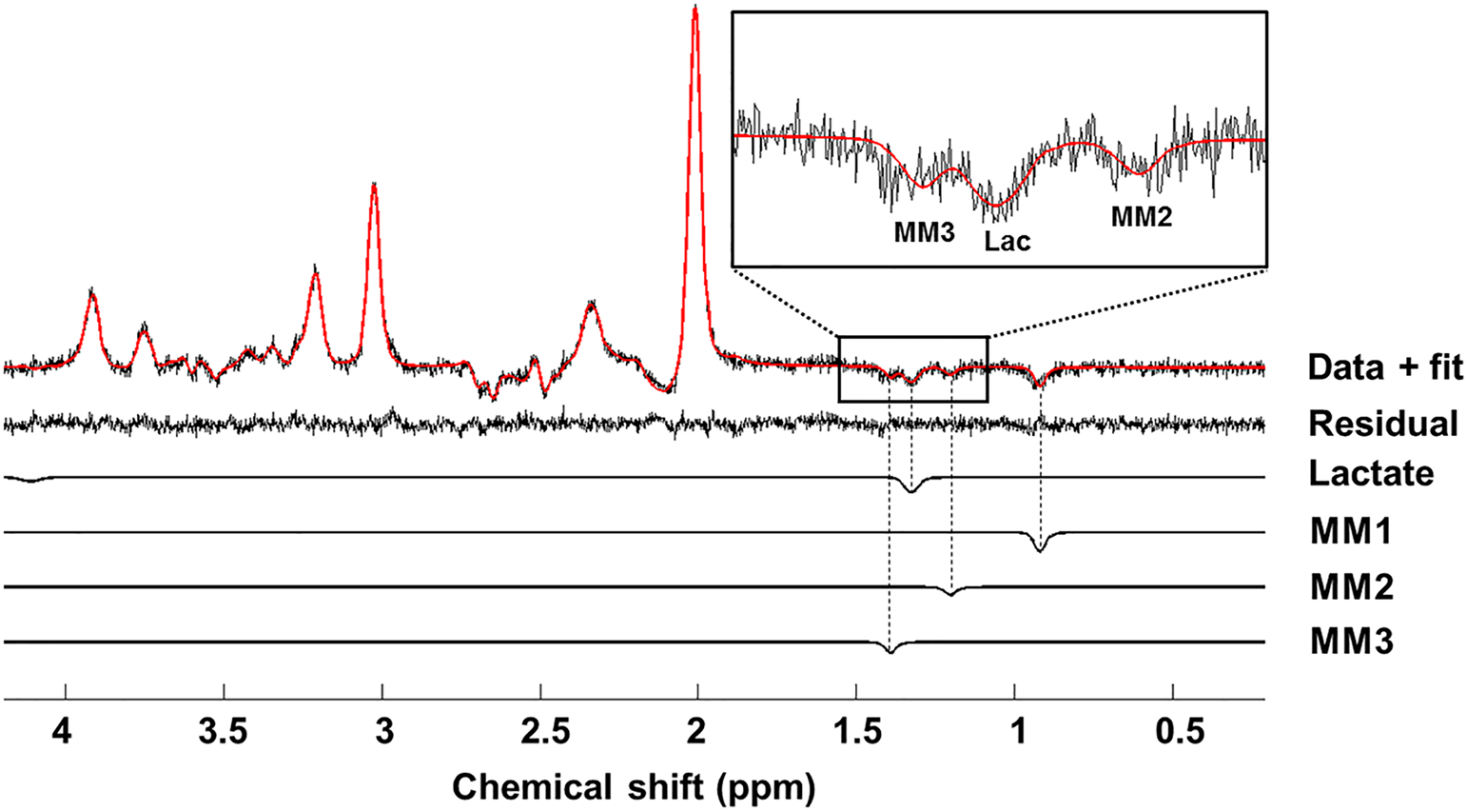
Goodness of fit. Example of an individual subject spectrum (semi‐LASER, TR/*TE* = 5000/144 ms, voxel size = $20\times 30\times 20{\mathrm{mm}}^{3}$, averages = 60), related to one time point of the time course analysis, and corresponding LCModel fits for Lac and macromolecules MM1, MM2 and MM3. A flat residual between the data and LCModel fit was consistently obtained

All spectra were checked for potential artefacts, lipid contamination or poor shimming. The mean water linewidth analysed from the unsuppressed water spectra was 11.4 ± 1.0 Hz and the SNR of the Cr peak at 3.02 ppm during the paradigm was 52.1 ± 5.6, for all individual subject time points (9 time points × 6 subjects). Figure 3B shows the individual subject spectra (overlaid in different colours) summed over the paradigm time course. There is excellent consistency between subjects and no indication of lipid contamination. Additionally, Glu and Lac levels were estimated in LCModel with an average Cramér‐Rao lower bound (CRLB) of 2% and 8% (min/max of 6%/11%), respectively (based on individual subject spectra with 60 averages; data from all subjects included). This demonstrates the robustness and efficacy of the data acquisition and post‐processing methods. The simulated basis set with the addition of the relevant MM resonances enabled a good fit to be achieved with the individual spectra, including a well‐fitted inverted Lac peak (Figure 4).

**Figure 5:**
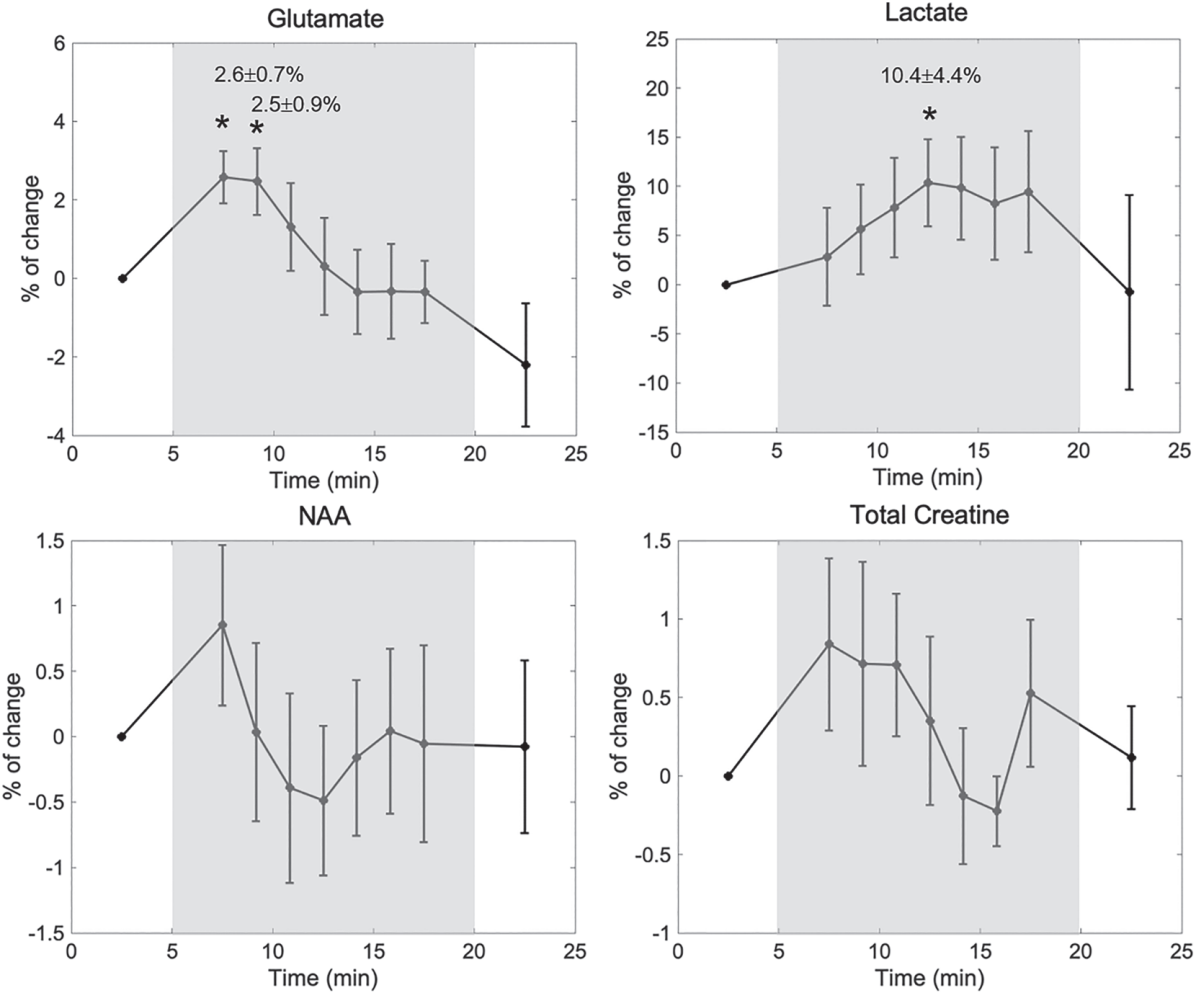
Analysis of all individual subject metabolic time courses. The mean percentage change relative to the metabolite concentration in the first OFF period was calculated from the quantification of individual subject spectra for Glu, Lac, NAA and total creatine. * indicates a significant difference ( P<0.05). Error bars represent the standard error of the mean. The ON period of the paradigm is shown as the shaded area

**Figure 6:**
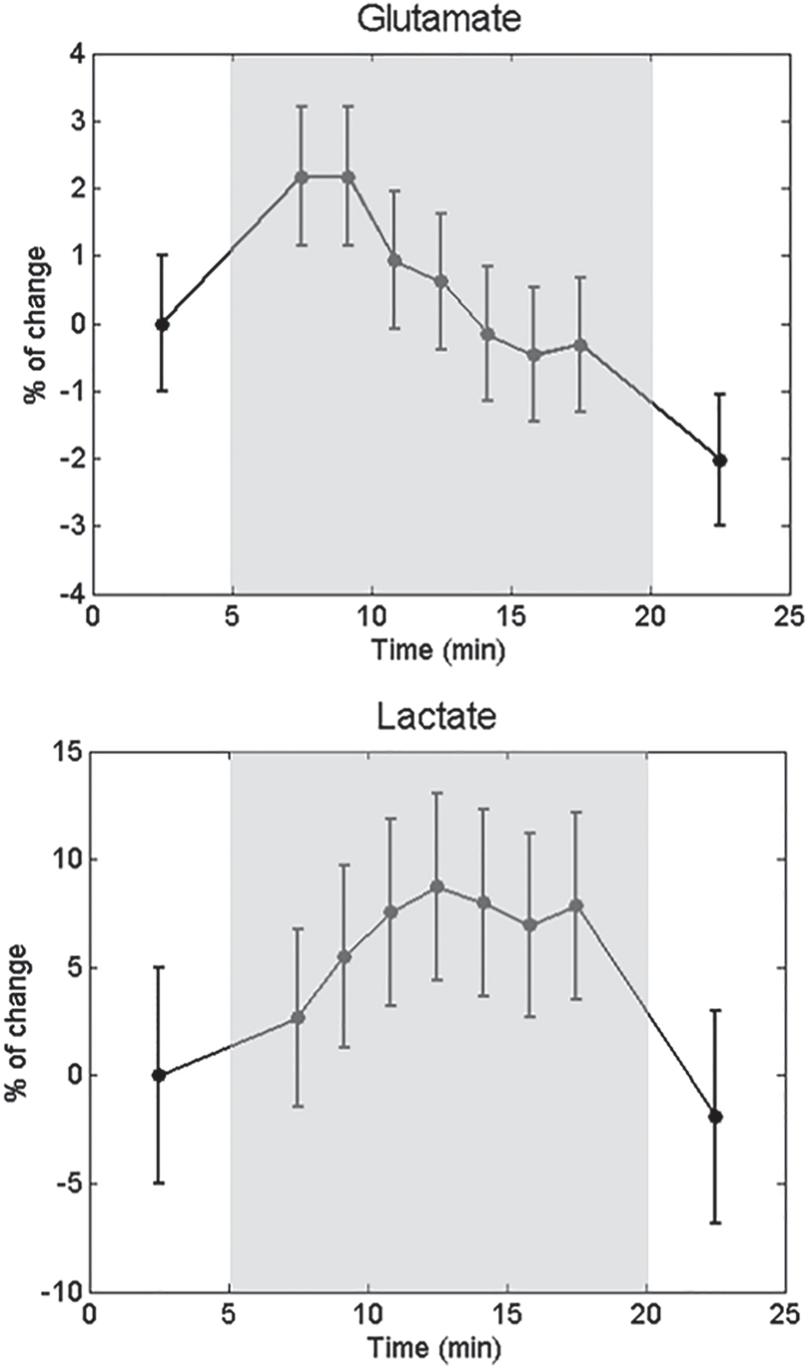
Analysis of group‐averaged metabolic time courses. Glu and Lac time courses during a paradigm, which consisted of 15 min of visual stimulation (shaded area), preceded and followed by 5 min of rest. The time courses were calculated on the group‐averaged spectra ( N=6) quantified using LCModel. The percentage change was determined based on the concentration during the first OFF period of the paradigm. The error bars represent the CRLBs of the spectral fitting

Changes in metabolite levels were expressed as percentage changes relative to baseline (first OFF period). Metabolic changes during the paradigm based on the analysis of all individual subject spectra are depicted in Figure 5. Significant ( P<0.05) increases in Glu levels of 2.6 ± 0.7% and 2.5 ± 0.9% were observed at 7.5 and 9.2 min, respectively, as an immediate response to the stimulus, as well as a rise in Lac levels of 10.4 ± 4.4% reached halfway through the stimulation. The analysis of the group spectra (Figure 6) confirmed the Glu and Lac response patterns obtained from the analysis of individual subject data. No statistically significant changes in NAA or total creatine levels were observed during the stimulation (Figure 5). The measured difference in linewidth between the first OFF period and the ON period was −0.10 ± 0.35 Hz and −0.11 ± 0.38 Hz for the NAA and Cr singlets, respectively.

## 4 DISCUSSION

A proper quantification of the key metabolites involved in neuronal activation is fundamental, in order to elucidate the mechanisms that support brain activity. This can be challenging, especially for Lac, whose signal is strongly contaminated by MM and lipid resonances; this contamination is particularly problematic when the selected VOI is in a region adjacent to pericranial fat, as in the case of the occipital cortex in fMRS studies with visual stimulation. In this work, we minimized the overlap of MM/lipids and Lac peaks by using a long TE semi‐LASER sequence in combination with saturation slabs. The semi‐LASER sequence is regularly employed in ultra‐high field studies for its low susceptibility to RF inhomogeneities and the optimized spatial selection properties of its refocusing pulses, which are essential in reducing the chemical shift displacement artefact and mitigating partial volume effects. We have used it with a *TE* of 144 ms in order to obtain an out‐of‐phase Lac peak, as well as to exploit the differences in $T_{2}$ relaxation times between metabolites and MM/lipids to achieve a cleaner baseline. The remaining MM signals (MM1‐3) were identified through visual inspection of metabolite‐nulled spectra, and were then individually fitted with simulated Lorentzian peaks. This enabled the variability seen in these components between subjects to be adequately accounted for, an advantage over short TE methods with a general measured MM profile. Thus, the results from this study show highly reproducible spectra obtained from different subjects, with no manifestation of artefacts or lipid contamination. Lac was precisely quantified in this study with an average CRLB of 8% (based on spectra with 60 averages). This is at the lower end of the range of previously reported values from 7 T fMRS studies using short TE sequences, which generally yield spectra with higher SNR (average CRLBs from 6% to 18%^6^, ^8^, ^9^, ^11^, ^12^). We attribute this low error to the improved fit arising from the clean baseline achieved around the Lac resonance at longer TE and the inclusion of individual MM components in the basis set. The analysis of the metabolite time courses of both individual subject spectra and the group‐averaged spectra revealed similar patterns. The Glu time course showed an increase in Glu levels within the first minutes of stimulation, followed by a return to baseline. In contrast, Lac levels gradually increased during the ON period, reaching a maximum in the middle of this period, and maintained a steady state until the end of the stimulation. The changes in Glu and Lac levels proved to be significant when compared with the first OFF period of each individual subject time course. The scale of these changes is consistent with previous observations performed at 7 T; the percentage change for Glu was within the reported range (2% to 5%), as was that for Lac (7% to 37%). The rapid rise of Glu levels (significant for the first two time points following the start of visual stimulation) probably does not reflect de novo synthesis of Glu/Gln but a change in the balance between them, as the Glu/Gln cycle rate increases during neural stimulation. Lin et al^7^ reported a 2% increase in Glu mirrored by an 8% fall in Gln, with no significant change in Glu + Gln in a similar visual activation paradigm, suggesting that this is the case. The failure to maintain a significant elevation of Glu concentration throughout the long stimulation period may be due to adaptation. In contrast, an increase in Lac concentration requires an increase in glycolytic flux, as there is no large pool of pyruvate from which it can be immediately derived. This is likely to take several minutes to reach a level that can be detected by ${}^{1}H$ MRS, and indeed this is what we observe. Interestingly, linewidth narrowing was not observed for NAA or Cr during visual stimulation. Previous work, which used short TE sequences, has shown that the linewidth of these peaks is reduced by approximately 0.4‐0.5 Hz at 7 T upon activation, due to the BOLD effect.^6^ The absence of BOLD effect observed in this study could be ascribed to changes in linewidth due to subtle deterioration in shimming occurring during the paradigm or the variability in the BOLD response within the limited number of subjects examined. Alternatively, the absence of an increase in $T_{2}^{*}$ could indicate that the signal changes arising from the BOLD effect are hidden during neuronal activation. Results from a functional diffusion‐weighted spectroscopy study^26^ suggest that the increase in the apparent diffusion coefficient of NAA and total creatine during visual stimulation could reflect a rise in intracellular molecular mobility or changes in neuronal microstructure. More recent work by the same group^27^ has demonstrated that both the apparent diffusion coefficient and the apparent $T_{2}$ of NAA, total creatine and Cho are dependent on the chosen TE. The explanation offered is that there are two (or more) pools of these metabolites: a more restricted fraction with a longer $T_{2}$ and a more mobile fraction with a shorter $T_{2}$. The contribution from the more mobile fraction would be selectively attenuated at longer TE values, and if, as seems likely, it is predominantly this fraction that is subject to the BOLD effect, may impact the linewidth change in our measurements. In conclusion, the purpose of this study was to evaluate acquisition and analysis strategies that should increase the sensitivity of Lac measurement, in order to provide a reliable method to investigate its role in neuronal activation. We successfully demonstrated the efficacy of this protocol, which compares favourably with the short TE approaches described in the literature. However, a systematic comparison between long and short TE methods to assess the precision and temporal resolution of Lac quantification with the long TE sequence in face of the inherent decrease in SNR is desirable. In addition, further interpretation of the metabolic changes and their significance in a long stimulation paradigm requires additional studies with larger sample size.
